# The Use of Traditional Chinese Medicine in Relieving EGFR-TKI-Associated Diarrhea Based on Network Pharmacology and Data Mining

**DOI:** 10.1155/2021/5530898

**Published:** 2021-04-01

**Authors:** Shuaihang Hu, Wenchao Dan, Jinlei Liu, Peng Ha, Tong Zhou, Xinyuan Guo, Wei Hou

**Affiliations:** ^1^Guang'anmen Hospital, China Academy of Chinese Medical Sciences, Beijing 100053, China; ^2^Beijing University of Chinese Medicine, Beijing 100029, China; ^3^University of Leicester, University Road, Leicester LE1 7RH, UK; ^4^Cancer Hospital Chinese Academy of Medical Sciences, Beijing 100021, China

## Abstract

In this study, the role of traditional Chinese medicine (TCM) in relieving epidermal growth factor receptor-tyrosine kinase inhibitor- (EGFR-TKI-) associated diarrhea was discussed by network pharmacology and data mining. Prediction of drug targets by introducing the EGFR-TKI molecular structures into the SwissTargetPrediction platform and diarrhea-related targets in the DrugBank, GeneCards, DisGeNET, and OMIM databases were obtained. Compounds in the drug-disease target intersection were screened by absorption, distribution, metabolism, and excretion parameters and Lipinski's rule in Traditional Chinese Medicine Systems Pharmacology. TCM-containing compounds were selected, and information on the property, taste, and meridian tropism of these TCMs was summarized and analyzed. A target-compound-TCM network diagram was constructed, and core targets, compounds, and TCMs were selected. The core targets and components were docked by AutoDock Vina (Version 1.1.2) to explore the target combinations of related compounds and evaluate the docking activity of related targets and compounds. Twenty-three potential therapeutic TCM targets for the treatment of EGFR-TKI-related diarrhea were obtained. There were 339 compounds acting on potential therapeutic targets, involving a total of 402 TCMs. The results of molecular docking showed good binding between the core targets and compounds, and the binding between the core targets and compounds was similar to that of the core target and the recommended drug loperamide. TCMs have multitarget characteristics and are present in a variety of compounds used for relieving EGFR-TKI-associated diarrhea. Antitumor activity and the efficacy of alleviating diarrhea are the pharmacological basis of combining TCMs with EGFR-TKI in the treatment of non-small-cell lung cancer. The core targets, compounds, and TCMs can provide data to support experimental and clinical studies on the relief of EGFR-TKI-associated diarrhea in the future.

## 1. Background

Primary bronchial lung cancer is the most common malignant tumor in the world, of which approximately 80–85% are non-small-cell lung cancer (NSCLC), and nearly 70% of NSCLC patients are in the locally advanced stage or have metastatic lesions at the primary diagnosis [1]. Although chemotherapy was once considered the first-line treatment for NSCLC, advancements in targeted gene research have given rise to epidermal growth factor receptor-tyrosine kinase inhibitors (EGFR-TKIs), such as erlotinib and gefitinib, to replace chemotherapy as the first-line therapy for NSCLC patients who are positive for the relevant driver genes [2, 3]. The adverse reactions to EGFR-TKI are different from those to chemotherapy, which include myelosuppression, nausea, and vomiting; the administration of EGFR-TKI is prone to causing rashes, diarrhea, and other adverse reactions, which have a negative impact on the patients' quality of life and can even lead to drug discontinuation. Therefore, it is essential to prevent and control adverse reactions during targeted therapy.

In clinical practice, common EGFR-TKI drugs are divided into three generations: the first generation includes gefitinib, erlotinib, and icotinib; the second includes afatinib; the third includes osimertinib. All these drugs can cause varying degrees of adverse reactions, and these common adverse reactions to EGFR-TKI treatment limit the application of EGFR-TKI; the mechanism by which these adverse reactions occur is not clear, but studies have shown that it is related to the secretion of chloride [4]. According to a previously published EGFR-TKI phase III clinical study, the overall incidence of diarrhea is 9.5–95.2%. EGFR-TKI treatment should be suspended if Grade 2 diarrhea lasts more than 48 hours, and for Grade 3 or higher diarrhea, EGFR-TKI treatment should be stopped until the grade of diarrhea is reduced to 1 or below. If symptoms are not relieved in 14 days, the administration of EGFR-TKI should be discontinued. Loperamide is often used in the clinical treatment of severe diarrhea, but there are few studies on the corresponding precautionary measures [5].

TCM has a long history of use for the treatment of diarrhea and has advantages in treating EGFR-TKI-related diarrhea, because besides relieving diarrhea, it can regulate the gastrointestinal function of patients, increase appetite, and prevent the reoccurrence of diarrhea [6]. TCM combined with targeted drugs can enhance efficacy, reduce toxicity, and reverse drug resistance, improving the quality of life for patients. The mechanism of action for TCM mainly involves the regulation of the immune function of lung cancer patients, thereby enhancing the migration of T cells. At the same time, it can inhibit the proliferation of tumor cells and induce tumor cell apoptosis [7, 8].

**Figure 1:**
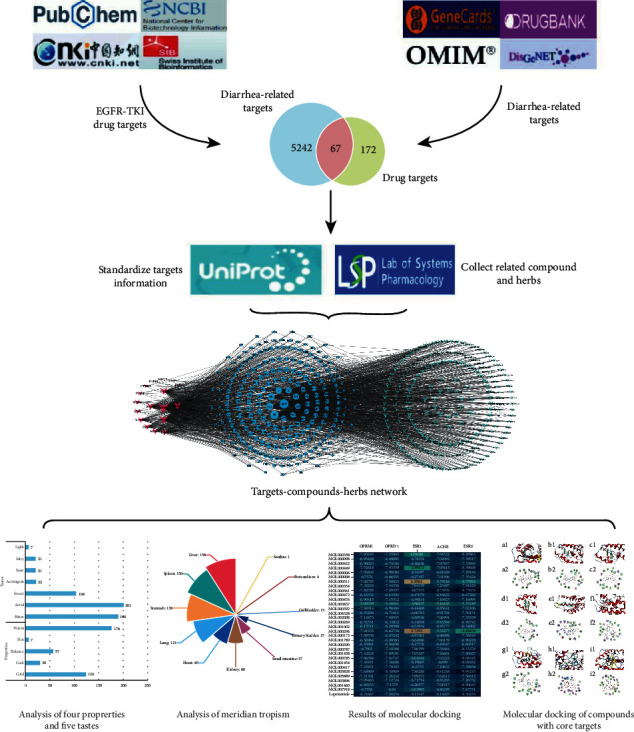
Framework of network pharmacology and data mining.

The synergistic effect of drugs on the human body can be explored through the analysis of network topology and nodes. Andrew Hopkins combined systems biology, pharmacology, information networks, and computer science to put forward network pharmacology, which demonstrates mutual complex disease-gene-target-drug relationships by applying a professional visual network analysis software in the form of network signals, allowing these relationships to be analyzed through the topology of the network and nodes [9]. Due to the pharmacodynamic material basis of TCM, the mechanisms and toxicological effects of compounds containing TCM are still not clear; as the quality of medicinal materials is difficult to control, it is different to study them on an integral level compared to a molecular level [10]. As TCM is characterized by “multiple components, multiple targets, low affinity, low selectivity”, and its effective constituents can be applied to various targeted diseases, it has obvious advantages in the treatment of multiple factors of a polygenic disease. Therefore, using network pharmacology to study multiple ways of regulating signaling pathways and to explain and explore how to effectively improve the efficacy of drugs is more in line with the ideals of TCM compounds. In this study, by screening drug and disease targets and using a variety of databases to screen compounds containing TCM, a target-compound-TCM relationship network was constructed. This network was then used to explore potential therapeutic targets and related compounds and identify TCMs that can relieve EGFR-TKI-associated diarrhea and have certain antitumor effects. These results can provide a scientific molecular basis for the clinical application of TCM in combination with EGFR-TKI. The specific process of the study is shown in Figure 1.

## 2. Materials and Methods

### 2.1. Screening of Drugs and Targets of the Disease

Through a literature search, the molecular formulae of gefitinib, erlotinib, icotinib, afatinib, and osimertinib were obtained. Their molecular structures were downloaded separately in SDF format using the PubChem database (pubchem.ncbi.nlm.nih.gov) and uploaded onto the SwissTargetPrediction (http://www.swisstargetprediction.ch) platform [11, 12], with species set to “*Homo sapiens*”, to predict the target of drug action. The results from the SwissTargetPrediction platform were supplemented by consulting the literature.

To obtain the targets of diseases related to diarrhea, a search through the GeneCards (http://www.genecards.org) [13], OMIM (omim.org) [14], DisGeNET (http://www.disgenet.org) [15], and DrugBank (http://www.drugbank.ca) [16] databases was performed using “diarrhea”, “diarrhoea”, and “diarrheas” as keywords. Drug and disease targets were converted into standard gene names through the UniProt database (https://www.uniprot.org/) [17]. The targets from the intersection of drug prediction targets and disease targets were obtained.

### 2.2. Screening Target-Related Compounds and Potential Therapeutic Targets

A search was conducted for the compounds related to the intersection targets through the database of Traditional Chinese Medicine Systems Pharmacology (TCMSP; http://tcmspw.com/index.php) [18] and filtered by absorption, distribution, metabolism, and excretion (ADME) parameters and Lipinski's rule [19–22]: oral bioavailability: ≥30%; drug-likeness: ≥0.18; the number of bonds which allow free rotation around themselves ≤ 10; topological polar surface area ≤60 Å^2^; and molecular weight: 180–500 Da.

As some of the data provided by the TCMSP database may be inconsistent with actual applications, compounds that were filtered out were checked one by one to supplement the related compounds. The screened compounds were mapped to the intersection targets of diseases and drugs, with targets not containing active ingredients excluded and potential therapeutic targets screened out, and a potential therapeutic target-compound network diagram was drawn using Cytoscape 3.7.2 [23]. Core therapeutic targets were screened out after performing an analysis of the characteristics of the network using a network analyzer.

### 2.3. Screening and Analysis of TCMs

The TCMSP database provided a list of TCMs that corresponded to the compounds, and the corresponding TCMs were screened out through the relevant compounds. To make the research results standardized and practical, TCMs not listed in the “Pharmacopoeia of the People's Republic of China 2015 Edition” [24] were excluded. According to the “Pharmacopoeia of the People's Republic of China 2015 Edition” and the textbook “Chinese Pharmacy” in the “Thirteenth Five-Year Plan” [25], this study carried out frequency analysis on the components containing TCM.

### 2.4. Construction of Target-Compound-TCM Network

A target-compound-TCM network from the potential therapeutic targets and the corresponding compounds, as well as the TCMs contained in the candidate compound, was constructed using the software Cytoscape 3.7.2, and the network characteristics were analyzed by a network analyzer. The degree value indicates the number of connections a particular target has. The more related compounds there are in a single TCM and the more related targets the related compounds act on, the higher the degree value, based on the core compounds and TCMs selected [26].

### 2.5. Molecular Docking between Target and Compound

To confirm the credibility of the interaction between the core targets and the core components in the target-compound-TCM network and obtain a new drug-target combination, we selected five targets with a high median degree value in the target-compound-TCM network as receptors and used the recommended therapeutic drug, loperamide, as the ligand for molecular docking [6].

The crystal structures of the five selected targets were obtained from the Protein Data Bank (http://www.rcsb.org), a protein crystal database, and saved in PDB format. The 3D chemical structures of the candidate compounds were downloaded from the PubChem database and saved in SDF format; the output documents were converted to PDB format for subsequent molecular docking. The coordinate documents of the receptor and ligand were prepared using AutoDock Tools 1.5.6, with the water molecules deleted from the ligand, the ligand and receptor separated, nonpolar hydrogen added, and the Gasteiger charge calculated after it was saved in PDBQT format. The potential core ligand was subjected to the treatment of energy minimization, obtaining the ligand atom type after calculation, and finally saved in PDBQT format. The AutoDock Vina software was used for the calculation of docking of semisoft molecules. Compared with AutoDock4, AutoDock Vina 1.1.2 adopts a complex gradient algorithm and multithreading technology and performs molecular docking scoring to evaluate the matching degree and activity between the target and the ligand. A docking score of < −4.25 indicates binding activity between the ligand and the target, a score of < -5.0 indicates better binding activity, and a score of < −7.0 indicates strong docking activity [27].

## 3. Results

### 3.1. Drug-Disease Target Intersection

The probability value obtained from the SwissTargetPrediction platform to predict drug effect targets represents the probability that the query molecule targets the protein. From a total of 239 drug targets, the top 100, as ranked by probability, were selected after those with a probability of 0 were removed, and 5 drug targets were obtained after eliminating duplicates.

**Figure 2:**
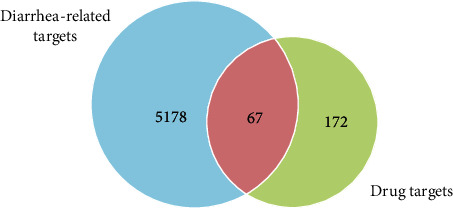
Intersection targets of EGFR-TKI and diarrhea.

A total of 5245 diarrhea-related disease targets were obtained by searching the disease databases, merging the data, and removing duplicate values. A total of 67 targets were obtained from the intersection of drug and disease targets, as shown in Figure 2.

### 3.2. Screening of Target-Related Compounds and Potential Therapeutic Targets

A total of 67 targets were obtained from the intersection of EGFR-TKI and diarrhea and imported into the TCMSP database; 29 targets and 834 compounds of relevance were retrieved. After the selection of the ADME parameters and Lipinski's rule, 293 compounds were obtained. The compounds excluded by the ADME parameters and Lipinski's rule were checked individually for the purpose of rigor, and 46 compounds associated with tumor treatment and adverse diarrhea effects were added, leading to a total of 339 compounds. There were 23 targets that met the 339 compounds at the intersection of EGFR-TKI and diarrhea; these were identified as the potential therapeutic targets for the treatment of adverse reactions of EGFR-TKI diarrhea with TCM (Table 1).

**Figure 3:**
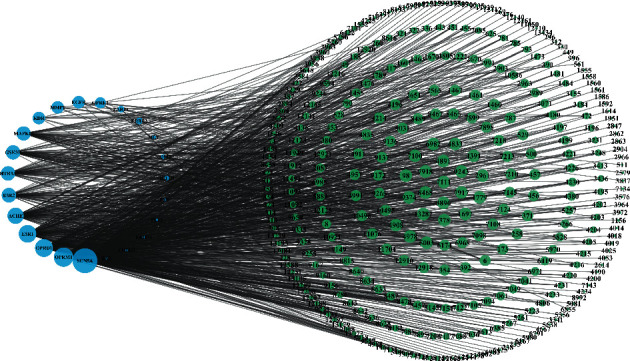
Target-compound network. (The network diagram consisted of 362 points and 1022 edges, with the potential therapeutic targets shown in blue and the compound targets shown in green.)

A network diagram of the final 339 compounds included with 23 potential therapeutic targets was constructed (Figure 3). To ensure a clear network diagram, the prefixes “MOL” and “0” of the compound name were deleted, leaving only the compound number. The network feature analysis was performed through a network analyzer plug-in, where the degree value determines the number of node connections and the role of the reaction nodes in the network is positively correlated with the target size in the diagram. Sodium channel protein type 5 subunit alpha (SCN5A), OPRM1, OPRD1, ESR1, ACHE, and ESR2 were core targets with large degree values of 280, 158, 128, 121, 90, and 55, respectively. The compounds with large degree values all had multiple potential therapeutic targets, suggesting that the selection of the compounds corresponded to the ideals of TCM and had extensive therapeutic value.

### 3.3. Compounds Corresponding to TCM

The final 339 compounds included in the network diagram were imported into the TCMSP platform to obtain 432 kinds of TCMs contained in the compounds. Following the deletion of 30 kinds of TCMs that were not recorded in the “Pharmacopoeia of the People's Republic of China 2015 Edition”, a total of 402 kinds of TCMs were incorporated.

**Figure 4:**
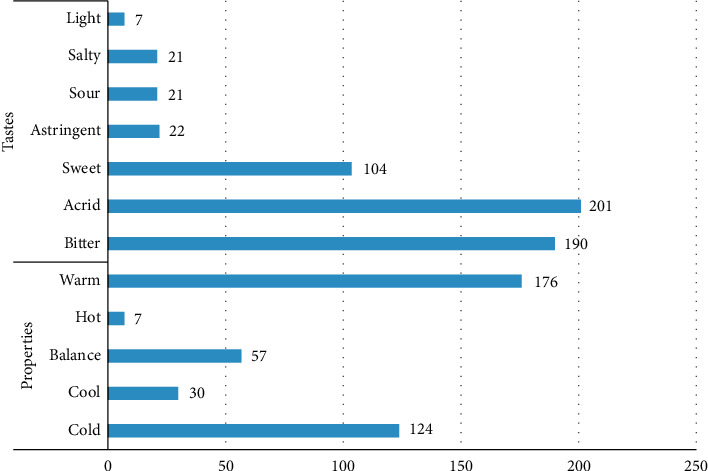
Analysis of four properties and five tastes.

**Figure 5:**
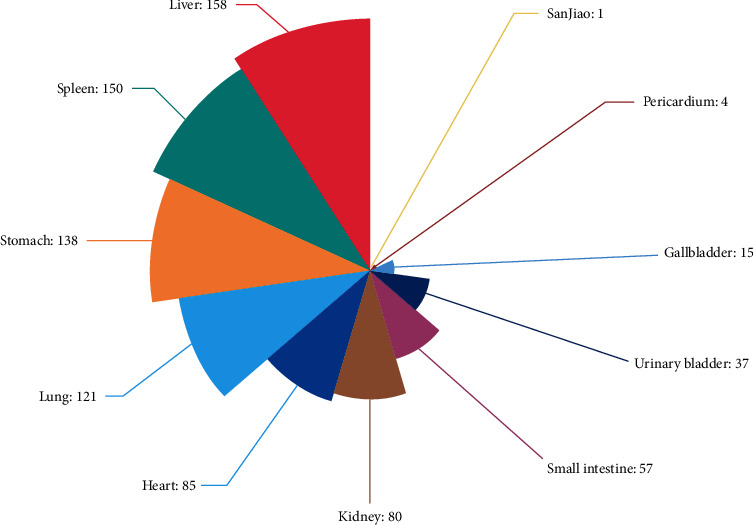
Analysis of meridian tropism.

The property, taste, and meridian tropism of the 402 TCMs were summarized and their frequency was analyzed. Acrid and bitter drugs had the highest frequencies, accounting for 35.51% and 33.56% of the selected TCMs, respectively. Drugs that were warm in property were the most frequently occurring, accounting for 44.67% of the selected TCMs, followed by those cold in property, which accounted for 31.47%. The frequency analysis of meridian tropism showed that the frequency of liver, spleen, and stomach was the highest in the included TCMs, accounting for 52.71% of the total, while 14.3% were attributed to the lungs. It is evident that most of the TCMs included were closely related to the liver, spleen, and stomach, followed by the lungs. These findings are consistent with the TCM theory of treating diarrhea and are related to the TCM theory of the digestive system (Figures 4 and 5).

### 3.4. Construction of the Target-Compound-TCM Network and Selection of Core Components

**Figure 6:**
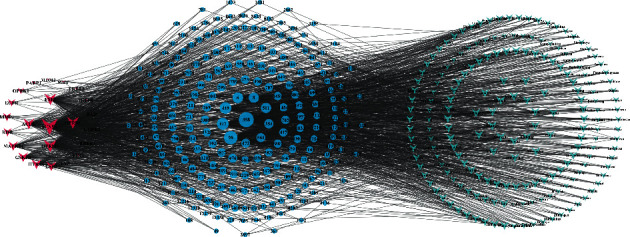
Target-compound-TCM network (compound degree ≥ 4; TCM degree ≥ 4; the network diagram consisted of 362 points and 1022 edges, with the potential therapeutic targets being shown in blue and the compound targets being shown in green).

A network diagram of 23 potential therapeutic targets, 339 compounds, and 402 kinds of TCMs was constructed, with 764 nodes and 2707 edges. For a clearer visual representation of the network relationships, only compounds and TCMs with degree values higher than the median (degree ≥ 4) were kept in the network (Figure 6).

The top 10 TCMs based on degree value in the target-compounds-TCM network diagram were Yanhusuo, Danshen, Gancao, Wuzhuyu, Huangbo, Guanhuangbo, Leigongteng, Baiqvcai, Gouteng, and Huangqi, as candidate compounds 38, 28, 26, 16, 16, 13, 13, 12, 12, and 11, respectively. Using the connection relationship between the related compounds and the targets in TCM, the potential targets of the TCMs for the treatment of EGFR-TKI-related diarrhea were collected. It was indicated that Yinyanghuo, Gaoliangjiang, Huangqi, Huzhang, Ziwan, Gancao, Jinqiaomai, Juhua, Yinxingye, and Heye were related to more targets, acting on 20, 18, 18, 18, 18, 17, 17, 17, 17, and 16 targets, respectively, showing that these TCMs had a strong modulating function on diarrhea as an adverse effect of EGFR-TKI. The core compounds were selected with a median degree value of 4 and a standard of 8, which was greater than twice the median of the degree value. The top 10 compounds based on the degree value of the compounds were *β*-sitosterol, quercetin, kaempferol, stigmasterol, luteolin, apigenin, ursolic acid, isorhamnetin, astragalin, and emodin (Table 2).

### 3.5. Molecular Docking Results

**Figure 7:**
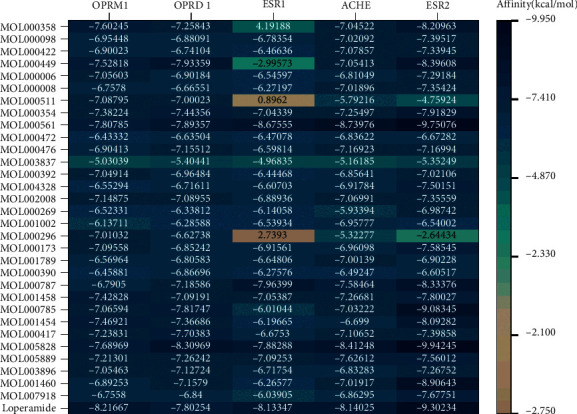
Results of molecular docking.

The molecular docking of five core targets, OPRM1 (PDBID: 4DKL), OPRD1 (PDBID: 4N6H), ESR1 (PDBID: 1A52), ACHE (PDBID: 1ODC), and ESR2 (PDBID: 1L2J), with 31 core compounds and the commonly used therapeutic drug loperamide, resulted in a total of 186 receptor-ligand docking results (Figure 7). There were 38 combinations in the target-compound network with the highest docking score of −9.9424 kcal/mol from MOL005828(nobiletin)-ESR2 and the lowest docking score of −6.0104 kcal/mol from MOL000785(astragalin)-ESR1. The average score of −7.0956 kcal/mol for the intranetwork combination implies good binding activity between the core targets and the core compounds, further proving the reliability of the therapeutic relationship between the compounds and the targets. Meanwhile, there were 117 new combinations outside the target-compound network. The top 3 combinations ranked according to affinity were MOL000561 (palmatine)-ESR2 with a score of −9.7507 kcal/mol, MOL001460(berberine)-ESR2 with a score of -8.9064 kcal/mol, and MOL000561(palmatine)-ACHE with a score of −8.7397. The average score of the off-net combination was −6.7672 kcal/mol. The docking results showed that there were still drug-target combinations that were not included in the TCMSP, and the effects of the compounds in TCM on the adverse reactions to EGFR-TKI in the form of diarrhea need to be further studied. The core targets and docking of loperamide can be summarized as follows: −8.2166 kcal/mol with OPRM1, −7.8025 kcal/mol with OPRD1, −9.3023 kcal/mol with ESR2, −8.1334 kcal/mol with ESR1, and −8.1402 kcal/mol with ACHE. This research has demonstrated that the potential therapeutic targets and drugs have good binding activity and have verified that the core targets selected in this study are of great significance for the treatment of diarrhea. The above-mentioned docking results offer reliable data support for further exploration of effective TCM ingredients and can serve as the theoretical reference for selecting effective targets and ingredients in future tests.

**Figure 8:**
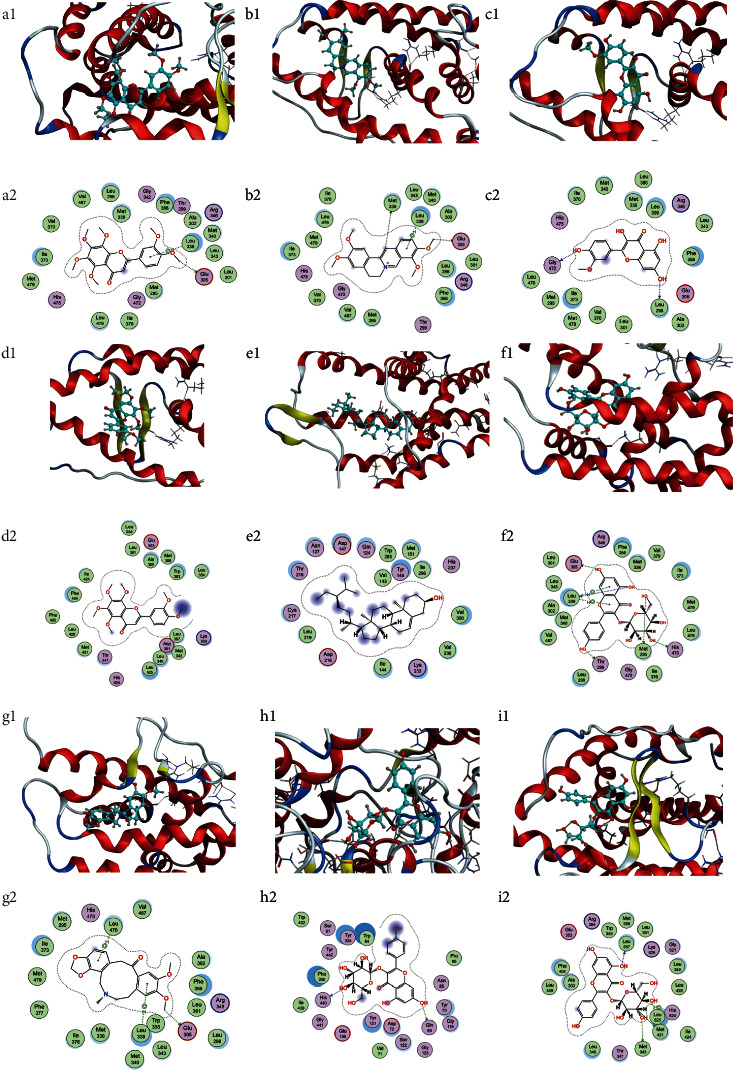
Molecular docking structure. (A) The overall 3D structure of the ligand-protein complex. The backbone of the protein was rendered as tubes and colored by chains. The ligands were rendered as sticks and colored in blue. (B) The 2D protein-ligand interaction diagrams of ligand-protein complexes. Protein residues were rendered as circles and colored based on their properties: green, hydrophobic residue; purple, polar residue (a: ESR2-nobiletin; b: ESR2-astragalin; c: ESR2-formononetin; d: ESR1-nobiletin; e: OPRM1-daidzein; f: ESR2-palmatine; g: ESR2-berberine; h: ACHE-palmatine; i: ESR1-palmatine).

Taking into account the affinity values for molecular docking and the degree values of the target-compound-TCM network, the docking patterns with affinity <−10 in the target-compound-TCM network and the top 4 ranked by affinity values outside the network were presented as 2D and 3D molecular docking pattern plots. As shown in Figure 8, each ligand was inserted into the active pocket of the target and reacted with a number of residues of the target via hydrophobic interaction and hydrogen bond formation.

## 4. Discussion

At present, for NSCLC patients who are positive for EGFR gene mutations, using EGFR-TKI as the first-line treatment leads to better progression-free survival, especially in the Asia-Pacific region and Russia, where EGFR gene mutations are present in 49.3% of the total number of patients with NSCLC [28, 29]. However, diarrhea is a common adverse event of EGFR-TKI drug use; if not taken correctly, it may lead to dehydration, forced reduction in drug dose, or even the interruption of treatment. The mechanism leading to such adverse reactions is not yet clear. It may involve imbalances in the ion transport system and abnormal secretion of chlorides, leading to secretory diarrhea [30], which lack effective preventive drugs. Although diarrhea is a common adverse reaction to all EGFR-TKIs, data show that second-generation inhibitors with a wider range of activities and targets cause a higher incidence of diarrhea [31]. Therefore, it is particularly important to explore the therapeutic targets related to EGFR-TKI and the drug-target relationship to investigate how EGFR-TKI may lead to adverse reactions such as diarrhea and to find effective therapeutic targets and drugs. Many studies have confirmed that TCM combined with EGFR-TKI treatment can improve the therapeutic effects and reduce toxicity by decreasing the incidence of diarrhea as an adverse reaction [32–34], indicating that TCM has certain advantages in the treatment of diarrhea. It can also enhance the therapeutic effects, but the research on related drugs and molecular mechanisms is still insufficient and lacks specific TCM intervention in studies of EGFR-TKI use with diarrhea as an adverse reaction. Therefore, it is of great clinical significance to assess effective therapeutic ingredients and targets in TCM. This research used network pharmacology to explore the mechanisms behind multicomponent and multitarget TCMs, to identify effective therapeutic targets, related compounds, and related TCMs, and to provide data support for using TCM to further improve the therapeutic effect of EGFR-TKI and reduce the incidence of diarrhea.

### 4.1. Core Targets

After screening the potential therapeutic targets, those with a high degree 294 value were screened for certain antitumor and antidiarrhea effects, such as OPRM1 and OPRD1. Opioids that act through such targets can reduce gastrointestinal propulsive peristaltic contractions and increase circular muscle tension and intraluminal pressure [35]. Endogenous opioids maintain gastrointestinal homeostasis. The activation of intestinal opioid receptors such as *μ* receptors and *δ* receptors reduces epithelial secretion and increases water/electrolyte absorption, producing an antidiarrheal effect [36]. As a synthetic opioid receptor agonist, loperamide, a recommended drug for the treatment of diarrhea as an adverse reaction to EGFR-TKI, not only acts through *μ* receptors in the muscular plexus of the intestinal wall but also inhibits the release of acetylcholine, thus reducing intestinal peristaltic activity [37]. Antidiarrheal drugs based on such targets are under continuous development [38, 39]. The neurotransmitter acetylcholine participates in various activities mainly through nicotinic and muscarinic receptors, of which M3 receptors are widely distributed in the gastrointestinal tract. It plays an important role in maintaining the contraction of gastrointestinal smooth muscle [40]. ACHE, which is one of the core therapeutic targets identified in this study, can convert acetylcholine to choline and acetic acid, thereby preventing the contraction effect of acetylcholine on the smooth muscles of the gastrointestinal tract and producing a specific antidiarrheal effect. This feature has the same effect as loperamide in inhibiting the release of acetylcholine.

ESR1 and ESR2 are estrogen receptors, and studies have confirmed that there is a significant correlation between ESR1 expression and the overall survival of patients with NSCLC. Estrogen-induced growth of transplanted cells in vitro correlated closely with acute hormonal activation of mitogen-activated protein kinase signaling, and treatment with the antiestrogen drug Faslodex can reduce its growth. ESR1 expression is considered an independent prognostic factor for metastatic NSCLC [41, 42]. The single nucleotide polymorphisms of ESR2 are closely related to lung cancer in nonsmoking women [43], and studies have shown that nonsmoking women in Asia have a higher mutation rate of the EGFR gene [44]. We believe that ESR2 is correlated with EGFR's effect on metabolism during the treatment of NSCLC. However, at present, there are few studies on this kind of research, and its mechanism is not clear.

SCN5A is mainly responsible for the initiation and propagation of cardiac action potentials, thereby generating cardiac excitability and electrical stimulation to the cardiac muscles. Dysfunction of this target will cause excess sodium to enter the cardiomyocytes through abnormal channels, which leads to long QT syndrome [45]. Related studies have shown that QT interval prolongation is a relatively rare adverse event of several TKIs such as osimertinib and crizotinib, and it is believed that the heart should be monitored during the treatment of axitinib in patients with a previous family history of heart disease [46].

Mok et al. observed advanced NSCLC patients with the EGFR T790M mutation after an EGFR-TKI treatment of oral axitinib plus cisplatin or carboplatin. Among them, 4% of patients taking osimertinib experienced QT interval prolongation, with 1% classified as having grade III adverse events [47]. At present, there are few studies on cardiotoxicity caused by TKIs. The SCN5A target identified in this study was predicted from the molecular structure of osimertinib on the SwissTargetPrediction platform. There were no such targets identified from other drug structures, so it is speculated that the adverse reaction of axitinib in prolonging QT interval may be related to this target, which provides new research ideas for the future exploration of TKI leading to adverse cardiac reactions.

### 4.2. Core Compounds

To better synergize with EGFR-TKI treatment, the selected compounds should not only alleviate the adverse reaction of diarrhea but also have an antitumor effect. Through topological analysis of the target-compound-TCM network, core compounds with higher degree values were identified and included *β*-sitosterol, quercetin, kaempferol, stigmasterol, luteolin, and apigenin. All of them have antitumor effects and can relieve the adverse reaction of diarrhea to a certain extent.

*β*-Sitosterol, which is widely present in various plants [48], can interfere with various cell signaling pathways and affect the cell cycle, thereby inhibiting tumor cell proliferation. It has anticancer properties against breast, prostate, colon, lung, and gastric cancers [49]. *β*-Sitosterol can also inhibit the growth of A549 cells in a dose- and time-dependent manner, arresting the cell cycle of the tumor cells in the G2/M phase. In vivo toxicity experiments show that *β*-sitosterol is nontoxic and reduces the absorption of cholesterol in the intestinal tract. It is a potential drug for the treatment of lung cancer [50].

Quercetin has various pharmacological properties such as having antitumor effects, conveying protection to the blood vessels, having anti-inflammatory and antioxidative properties, and being involved in immune regulation. Its main antitumor mechanism is related to the induction of apoptosis by inhibiting the cellular signal proteins regulated by antiapoptotic proteins, such as B-cell lymphoma-2 (Bcl-2), and upregulating proapoptotic proteins. It also inhibits cell proliferation by blocking the progression of the cell cycle from G0/G1 to G2/M and inhibits angiogenesis and metastasis [51]. A study has shown that quercetin promotes the apoptosis of A549 cells by downregulating the expression of matrix metallopeptidase 9 (MMP-9) and transforming growth factor-*β* (TGF-*β*) [52]. Li et al. [53] used various concentrations of quercetin and gemcitabine to treat lung cancer alone or in combination and found that quercetin, when combined with gemcitabine treatment, could act as a chemosensitizer by downregulating heat shock protein 70 (HSP70). However, its poor water solubility and low bioavailability limit further clinical use [54]. Phycophyllin also has good antitumor activity, and as the most active antidiarrheal ingredient in guava, it can inhibit the invasion of gastrointestinal diseases caused by *Escherichia coli* and *Shigella flexneri* in HEp-2 cells. Its anti-inflammatory and antioxidative effects can eliminate oxygen free radicals in the intestinal tract, reduce the loss of intestinal water and electrolytes, inhibit intestinal peristalsis caused by inflammation, and restore normal intestinal mechanical activity [55, 56].

Kaempferol can block the cell cycle through a variety of mechanisms, thus inhibiting the proliferation of cancer cells. It can inhibit the concentration of cyclin-dependent kinase (CDK), arresting cells in the G2/M phase [57]. The overexpression of the cell myelomatosis virus oncogene (cMyc) in most cancers leads to excessive cell proliferation. Kaempferol can reduce the level of the cMyc mRNA and increase the level of the CDKN1A mRNA in cancer cells. The combination of cyclin-dependent kinase inhibitor 1A (CDKN1A) and the CDK complex inactivates CDK and blocks the cell cycle [58]. Kaempferol inhibits the secretion of vascular endothelial growth factor (VEGF) and inhibits angiogenesis [59]. Kaempferol and quercetin have synergistic antiproliferation effects on cancer cells through the decrease of the nuclear proliferating antigen Ki67 [60]. Inflammation is closely related to the occurrence and development of acute and chronic diarrhea. Kaempferol can regulate the activity of proinflammatory enzymes and inhibit transcription factors, adhesion molecules, and inhibit the expression of proliferation-related genes. At present, as the mechanisms behind diarrhea caused by EGFR-TKI are not clear, kaempferol, with the antitumor and anti-inflammatory properties, can provide a reference for the development of new drugs to alleviate the adverse reaction of diarrhea and strengthen EGFR-TKI therapy [61].

The other core components, such as stigmasterol, luteolin, apigenin, and ursolic acid, also have antitumor and anti-inflammatory effects. Stigmasterol inhibits cancer cell metastasis and arrests cell cycle in the G2/M phase [62]. It has also been shown to significantly decrease the expression of cyclooxygenase-2 (COX-2) and colony-stimulating factor-1, which could reduce the severity of colitis [63]. Luteolin can effectively inhibit the proliferation of NSCLC A549 cells by inducing apoptosis and inhibiting proinflammatory cytokines (IL-1*β*, IL-6, IL-8, and TNF-*α*) and VEGF, thus inhibiting angiogenesis [64]. A study has shown that luteolin has significant antitumor effects on EGFR with the L858R/T790M mutation and erlotinib-corrected NSCLC at the organism and cellular levels [65]. Luteolin is also a good antioxidant, scavenging free radicals and producing an anti-inflammatory effect [66]. Apigenin and ursolic acid are also good anti-inflammatory and antitumor drugs. Ursolic acid and its derivatives can induce tumor cell apoptosis, inhibit angiogenesis, tumor cell invasion, and metastasis, and can exert an anti-inflammatory effect by inhibiting COX-2 [67]. Apigenin can block the cell cycle in the G1/S and G2/M phases, induce apoptosis, and has anti-inflammation and antioxidation properties [68]. It can inhibit the proliferation of A549 lung cancer cells and the transcriptional activation of VEGF, thus inhibiting tumor neovascularization [69].

At present, the mechanism behind diarrhea as an adverse reaction to EGFR-TKI is not clear. However, some studies have suggested that diarrhea is caused by the decrease of intestinal epithelial growth and repair due to the inhibition of signal transduction in intestinal epithelial cells by EGFR-TKI [70]; EGFR-TKI causes the toxicity of the intestinal epithelium and intestinal inflammation, which in turn can increase the exfoliation and apoptosis of the intestinal epithelium [71]. The core compounds identified in this study include several flavonoids, such as quercetin, kaempferol, luteolin, and apigenin. As effective antioxidants, flavonoids have good anti-inflammatory and anticancer activities. Some studies have confirmed a correlation between the intake of flavonoids and lung cancer [72]. Therefore, the core compounds identified in this study can be used as potential therapeutic drugs for lung cancer, and their anti-inflammatory activities can provide a pharmacological reference for alleviating the adverse reaction of diarrhea to EGFR-TKI.

### 4.3. Molecular Docking

The results of molecular docking activity showed that in the target-compound-TCM network, the highest binding activity was between nobiletin and ESR2. Nobiletin, as a flavonoid compound contained in the TCM citrus peel, has good anticancer and antioxidant pharmacological effects. Nobiletin and its main metabolites, such as 4′-demethylnobiletin and 3′-desmethylnobiletin, can significantly inhibit the occurrence of lung cancer in mice by blocking the cell cycle and inducing apoptosis [73]. Nobiletin combined with paclitaxel, carboplatin, and other chemotherapeutic drugs can significantly inhibit the growth of subcutaneous A549 tumor xenotransplants in mice and enhance the sensitivity of A549 cells to chemotherapeutic drugs by regulating various signaling pathways such as the Akt/GSK3*β*/MYCN pathway [74, 75]. Nobiletin's good anti-inflammatory activity can alleviate the occurrence of diarrhea. Related studies have shown that nobiletin can protect the intestinal epithelium barrier by reducing inflammatory cytokines and can reduce duodenal retraction to treat inflammatory bowel disease by inhibiting tumor necrosis factor-*α* (TNF-*α*) and the expression of COX-2. It thus has potential intestinal protective function [76, 77].

In the past, there were few studies on the relationship between ESR2 and lung cancer. It is also widely believed that ESR2 is closely related to the occurrence and development of breast cancer [78]. However, studies have also found that ESR2 may be a new therapeutic target for lung adenocarcinoma in the future, as its downregulation reduces matrix metallopeptidase 2 (MMP-2) and MMP-9 expression, inhibiting the progression of lung adenocarcinoma through the MEK/ERK signaling pathway [79]. From the above, it can be seen that the targets and related compounds identified in this study have antitumor effects and can relieve the adverse reaction of diarrhea.

The target-compound-TCM network contains 117 target and compound docking combinations, including many compounds with good pharmacological activity, such as palmatine and berberine, which have high docking scores. Palmatine has anticancer, antioxidant, anti-inflammatory, neuroprotective, antibacterial, antiviral, and other pharmacological effects [80]. Berberine is used clinically to treat a variety of diseases with good pharmacological activity; it not only inhibits the proliferation, invasion, and metastasis of cancer cells but also has an excellent inhibitory effect on toxins and bacteria, including *Helicobacter pylori*. Its ability to protect the intestinal epithelial barrier from injury is used in the treatment of a variety of digestive system diseases [81]. The results of molecular docking showed that the selected compounds and the drug loperamide had good docking activity with their targets, with many compounds having a docking score higher than that of loperamide. It showed that the compounds selected in this study have good medicinal reference value, and the results of molecular docking can provide related compound and target references for the future study of good therapeutic drugs that can be combined with EGFR-TKI treatment.

### 4.4. Core Chinese Medicine

The core Chinese medicines identified through network pharmacology and data mining were mainly acrid and warm, and their meridian tropism mostly covered the liver, spleen, stomach, and lungs. The characteristics of these core Chinese medicines reflect the characteristics of the TCM theory for treating diarrhea by “warming yang and transforming dampness”. The theory of TCM believes that diarrhea originates in the spleen, and the transportation of the water in one's diet cannot be separated from the rise and fall of the spleen and stomach and the adjustment of liver gas. The location of lung cancer is mainly the lungs, and the meridian tropism of these core Chinese medicines aligns with the treatment theory of TCM, which provides a theoretical reference for clinical treatment with TCM.

The core TCMs obtained in this study were Yanhusuo, Danshen, Gancao, Wuzhuyu, Huangbo, Yinyanghuo, Gaoliangjiang, and Huangqi. Modern pharmacology has shown that Yanhusuo extracts contain 13-methyl-palmatrubine, which has antitumor activities and can inhibit the proliferation of lung cancer A549 cells by blocking the activation of the EGFR and MAPK signaling pathways [82]. Studies have confirmed that Yanhusuo alkaloid extracts can inhibit the VEGF signaling pathways, thereby producing antiangiogenic effects, and are expected to be used as antiangiogenic drugs [83]. Yanhusuo extracts also contain berberine, which not only inhibits the proliferation of cancer cells [84] but also can be used in the treatment of inflammatory bowel disease by protecting the intestinal epithelial barrier, regulating the transcription of intestinal inflammatory cytokines, and reducing proinflammatory cytokines such as TNF-*α*, IL-13, IL-6, IL-8, and IFN-*γ* [85]. Therefore, the core Chinese medicine Yanhusuo, when combined with EGFR-TKI treatment, not only has an antitumor effect but also reduces the incidence of diarrhea.

Other selected Chinese medicines also have such effects. The extract of Danshen contains cryptotanshinone, which can inhibit the proliferation of NSCLC cells through the miR-146a-5p/EGFR axis, block the G0/G1 phase of the cell cycle, and promote apoptosis [86, 87]. Tanshinone IIA combined with cisplatin treatment for NSCLC can prevent cancer cell migration and invasion, block the cell cycle in the S phase, and induce A549 cell apoptosis [88]. Gancao, as a widely used TCM, contains a variety of flavonoid compounds and has a variety of pharmaceutical effects, such as antioxidative, anti-inflammatory, antibacterial, antitussive, antiviral, anticancer, and antimutagenic effects [89]. Evodiamine, found in the extract of Wuzhuyu, can inhibit the proliferation of cancer cells through various methods, such as blocking the cell cycle and inhibiting the proliferation of A549 cells. It can induce protective autophagy inf Lewis lung carcinoma cells and has good anticancer activity against many kinds of cancer cells [90]. Evodiamine can also effectively inhibit excessive gastrointestinal motility, providing a basis for the treatment of adverse reactions of diarrhea [91]. In summary, the core Chinese medicines screened in this study are closely related to antitumor effects and can alleviate adverse reactions. Their good pharmacological activity provides a reference for the mining and utilization of related TCMs and provides a theoretical basis for related experimental research and the better use of TCM combined with EGFR-TKI in the treatment of NSCLC in the future.

## 5. Conclusion

In this study, using network pharmacology and data mining, the role of TCM in alleviating EGFR-TKI-associated diarrhea was explored; core targets, compounds, and TCMs were screened using the relevant topological analysis parameters, and the pharmacological activities of the included targets and compounds were further evaluated by molecular docking. Core targets such as OPRM1, OPRD1, and ESR1 were screened, and the related targets of rare adverse reactions such as SCN5A were identified. The core components selected included *β*-sitosterol, quercetin, kaempferol, and stigmasterol. The core TCMs selected included Yanhusuo, Danshen, Gancao, and Wuzhuyu. Not only were the relevant targets and compounds consistent with existing research, but also the meridian tropism covered by the core TCMs selected was consistent with the theory of TCM. These results can provide data support for the study of the antitumor effect of TCMs and their potential in alleviating diarrhea as an adverse reaction to EGFR-TKI treatment in the future and provide a basis for exploring TCM combined with EGFR-TKI in the treatment of NSCLC.

The advantage of this study is that the relevant data were obtained from an authoritative database. However, a disadvantage is that some of the data may be outdated. Another disadvantage is that the research results depend on the calculation of the software used. There are also certain limitations as the contents of each component of the TCMs are not exactly defined. The results still need to be verified by subsequent animal and clinical trials.

## Figures and Tables

**Table 1:** Potential therapeutic targets of TCM for the treatment of EGFR-TKI-related diarrhea.

ID GeneSymbol	Uniprot ID	Protein name
1 SCN5A	Q14524	Sodium channel protein type 5 subunit alpha
2 PARP1	P09874	Poly [ADP-ribose] polymerase 1
3 OPRM1	P35372	Mu-type opioid receptor
4 OPRK1	P41145	Kappa-type opioid receptor
5 OPRD1	P41143	Delta-type opioid receptor
6 MMP1	P03956	Interstitial collagenase
7 MET	P08581	Hepatocyte growth factor receptor
8 MDM2	Q00987	E3 ubiquitin-protein ligase Mdm2
9 MAPK14	Q16539	Mitogen-activated protein kinase 14
10 KDR	P35968	Vascular endothelial growth factor receptor 2
11 IGF1R	P08069	Insulin-like growth factor 1 receptor
12 HTR3A	P46098	5-Hydroxytryptamine receptor 3A
13 GSK3B	P49841	Glycogen synthase kinase-3 beta
14 FYN	P06241	Tyrosine-protein kinase fyn
15 FLT1	P17948	Vascular endothelial growth factor receptor 1
16 ESR2	Q92731	Estrogen receptor beta
17 ESR1	P03372	Estrogen receptor
18 ERBB3	P21860	Receptor tyrosine-protein kinase erbB-3
19 ERBB2	P04626	Receptor tyrosine-protein kinase erbB-2
20 EGFR	P00533	Epidermal growth factor receptor
21 CHEK2	O96017	Serine/threonine-protein kinase Chk2
22 BAD	Q92934	Bcl2-associated agonist of cell death
23 ACHE	P22303	Acetylcholinesterase

**Table 2:** Core compounds (degree ≥ 8).

MolID	MolName	CAS	Degree
MOL000358	Beta-sitosterol	83-46-5	234
MOL000098	Quercetin	117-39-5	185
MOL000422	Kaempferol	520-18-3	128
MOL000449	Stigmasterol	83-48-7	124
MOL000006	Luteolin	491-70-3	91
MOL000008	Apigenin	520-36-5	78
MOL000511	Ursolic acid	77-52-1	77
MOL000354	Isorhamnetin	480-19-3	43
MOL000561	Astragalin	480-10-4	36
MOL000472	Emodin	518-82-1	32
MOL000476	Physcion	521-61-9	30
MOL003837	Esculetin	305-01-1	26
MOL000392	Formononetin	485-72-3	23
MOL004328	Naringenin	153-18-4	23
MOL002008	Myricetin	529-44-2	22
MOL000269	Elemicin	487-11-6	20
MOL001002	Ellagic acid	476-66-4	18
MOL000296	Hederagenin	465-99-6	17
MOL000173	Wogonin	632-85-9	15
MOL001789	Isoliquiritigenin	961-29-5	15
MOL000787	Fumarine	130-86-9	14
MOL000390	Daidzein	486-66-8	14
MOL001458	Coptisine	3486-66-6	13
MOL000785	Palmatine	3486-67-7	12
MOL001454	Berberine	633-66-9	12
MOL005828	Nobiletin	478-01-3	11
MOL005889	Rhamnetin	90-19-7	11
MOL000417	Calycosin	20575-57-9	11
MOL003896	7-Methoxy-2-methyl isoflavone	19725-44-1	10
MOL001460	Cryptopin	482-74-6	9
MOL004891	Shinpterocarpin	157414-04-5	9
MOL007918	2-Hydroxy-7-methoxy-1,8-dimethyl-5-ethenyl-9,10-dihydrophenanthrene	—	9
MOL008468	Methyl (E)-2-[(2S, 3Z, 12bS)-3-ethylidene-2,4,6,7,12,12b-hexahydro-1h-indolo[3,2-h]quinolizin-2-yl]-3-methoxyprop-2-enoate	—	9
MOL000500	Vestitol	20879-05-4	8
MOL001461	Dihydrochelerythrine	6880-91-7	8

## Data Availability

The datasets supporting the conclusions of this study are included within the article.
